# Synchronous versus sequential chemo-radiotherapy in patients with early stage breast cancer (SECRAB): A randomised, phase III, trial

**DOI:** 10.1016/j.radonc.2019.10.014

**Published:** 2020-01

**Authors:** Indrajit N. Fernando, Sarah J. Bowden, Kathryn Herring, Cassandra L. Brookes, Ikhlaaq Ahmed, Andrea Marshall, Robert Grieve, Mark Churn, David Spooner, Talaat N. Latief, Rajiv K. Agrawal, Adrian M. Brunt, Andrea Stevens, Andrew Goodman, Peter Canney, Jill Bishop, Diana Ritchie, Janet Dunn, Christopher J. Poole, Daniel W. Rea, on behalf of the SECRAB Investigators

**Affiliations:** a Cancer Centre, University Hospitals Birmingham NHS Foundation Trust, Birmingham, United Kingdom; b Cancer Research UK Clinical Trials Unit (CRCTU), University of Birmingham, United Kingdom; c Leicester Clinical Trials Unit, University of Leicester, Leicester General Hospital, Leicester, United Kingdom; d Warwick Clinical Trials Unit, University of Warwick, Coventry, United Kingdom; e Oncology Unit, University Hospitals Coventry and Warwickshire NHS Trust, University Hospital, Coventry, United Kingdom; f Clinical Oncology, Worcestershire Royal Hospital, Worcester, United Kingdom; g The Shrewsbury and Telford NHS Trust, Royal Shrewsbury Hospital, Shrewsbury, United Kingdom; h Cancer Centre, Royal Stoke University Hospital & Keele University, Stoke-on-Trent, United Kingdom; i Oncology Unit, Torbay and South Devon NHS Foundation Trust, Torbay Hospital, Torquay, United Kingdom; j Beatson West of Scotland Cancer Centre, Glasgow, United Kingdom; k North Wales Cancer Treatment Centre, Glan Clwyd Hospital, United Kingdom

**Keywords:** Breast cancer, Radiotherapy, Chemo-radiotherapy, Clinical trial

## Abstract

**Background:**

The optimal sequence of adjuvant chemotherapy and radiotherapy for breast cancer is unknown. SECRAB assesses whether local control can be improved without increased toxicity.

**Methods:**

SECRAB was a prospective, open-label, multi-centre, phase III trial comparing synchronous to sequential chemo-radiotherapy, conducted in 48 UK centres. Patients with invasive, early stage breast cancer were eligible. Randomisation (performed using random permuted block assignment) was stratified by centre, axillary surgery, chemotherapy, and radiotherapy boost. Permitted chemotherapy regimens included CMF and anthracycline-CMF. Synchronous radiotherapy was administered between cycles two and three for CMF or five and six for anthracycline-CMF. Sequential radiotherapy was delivered on chemotherapy completion. Radiotherapy schedules included 40 Gy/15F over three weeks, and 50 Gy/25F over five weeks. The primary outcome was local recurrence at five and ten years, defined as time to local recurrence, and analysed by intention to treat.

ClinicalTrials.gov NCT00003893.

**Findings:**

Between 02-July-1998 and 25-March-2004, 2297 patients were recruited (1150 synchronous and 1146 sequential). Baseline characteristics were balanced. With 10.2 years median follow-up, the ten-year local recurrence rates were 4.6% and 7.1% in the synchronous and sequential arms respectively (hazard ratio (HR) 0.62; 95% confidence interval (CI): 0.43–0.90; *p* = 0.012). In a planned sub-group analysis of anthracycline-CMF, the ten-year local recurrence rates difference were 3.5% versus 6.7% respectively (HR 0.48 95% CI: 0.26–0.88; *p* = 0.018). There was no significant difference in overall or disease-free survival. 24% of patients on the synchronous arm suffered moderate/severe acute skin reactions compared to 15% on the sequential arm (*p* < 0.0001). There were no significant differences in late adverse effects apart from telangiectasia (*p* = 0.03).

**Interpretation:**

Synchronous chemo-radiotherapy significantly improved local recurrence rates. This was delivered with an acceptable increase in acute toxicity. The greatest benefit of synchronous chemo-radiation was in patients treated with anthracycline-CMF.

**Funding:**

Cancer Research UK (CR UK/98/001) and Pharmacia.

## Highlights

• SECRAB is the largest chemo-radiotherapy trial to date in early breast cancer.
• SECRAB showed a positive therapeutic benefit of using adjuvant synchronous chemo-radiotherapy.
• Synchronous chemo-radiotherapy should be used for ≤ 3 week radiotherapy schedules.
• SECRAB results are applicable to patients having CMF or anthracycline-CMF schedules.
• Overall survival outcome may increase for patients receiving synchronous anthracycline-CMF.

Standard of care when combining adjuvant treatment in operable early stage breast cancer is surgery with sequential chemotherapy (CT) followed by radiotherapy (RT) [1]. However, the optimal integration of adjuvant treatment remains controversial [2], [3], [4].

Recht et al. (1996) randomised 244 patients after breast conserving surgery to receive radiotherapy before or after chemotherapy [5]. Five year actuarial cancer recurrence rates suggested that delaying chemotherapy until after radiotherapy may result in an increased rate of distant metastases. Conversely the study showed that delaying radiotherapy until after chemotherapy completion leads to a higher risk of local recurrence (5% RT-CT versus 14% CT-RT). These results suggested there may be an advantage in giving synchronous chemo-radiotherapy as it would avoid delaying either treatment and may shorten overall treatment duration, but risks enhancement of both acute and late radiotherapy toxicities. However, in an update to the Recht et al., study, published after SECRAB commenced, the initial differences observed were no longer statistically significant between the chemotherapy-first and radiotherapy-first arms after longer follow up (135 months) [6].

It is worthwhile noting that the addition of anthracycline chemotherapy to adjuvant regimens was not routine practice in the United Kingdom (UK) when SECRAB was conceived in the mid-1990s. This subsequently became routine practice with a moderate improvement in overall survival [7]. Care must be taken when giving anthracyclines synchronously with radiotherapy due to worsening of acute and late radiotherapy toxicity [8].

Three randomised controlled trials have subsequently investigated synchronous versus sequential chemo-radiotherapy delivery and demonstrate mixed results varying from no benefit to a significantly improved five year loco-regional relapse free advantage in axillary node positive patients [9], [10], [11]. All three studies utilised a five week radiotherapy fractionation schedule. The ARCOSEIN trial randomised 716 patients to receive chemotherapy followed by radiotherapy or synchronous chemo-radiotherapy and showed a small advantage in node positive patients in five-year loco-regional relapse free survival in the synchronous arm (synchronous arm: 97% (7/154 recurrences); sequential arm: 91% (17/191 recurrences); *p* = 0.02) [9]. The study by Rouesse et al., also showed a borderline statistically significant difference in local recurrence rates at five years (synchronous arm: 3% (9/324 local recurrences); sequential arm: 7% (20/314 local recurrences); *p* = 0.047) [10]. However in both these studies there were significantly increased toxicities in the synchronous arm both in acute skin reaction and late effects such as fibrosis, breast shrinkage, telangiectasia, and oesophagitis. Limitations in these studies included small numbers of patients recruited and variation in chemotherapy regimens utilised in each arm, particularly with the use of mitoxantrone which is no longer used in adjuvant therapy due to the increased rate of leukaemia. The third study, using CMF chemotherapy, recruited 206 patients and reported five local recurrences in each arm [11]. Toxicity was not reported in the initial publication but a later retrospective assessment reported a four-fold increase in the odds of grade 2/3 fibrosis and breast retraction in the synchronous arm with no effect on telangiectasia [12]. There were mixed findings with regard to cosmesis [9], [10], [12].

The aims of the SECRAB trial were to establish whether synchronous chemo-radiotherapy improves local recurrence and whether this can be delivered with acceptable toxicity.

## Methods

### Study design

This pragmatic randomised controlled, open-label phase III trial was conducted in 48 centres in the UK and patients were recruited by 63 consultants. The protocol and subsequent amendments were approved by the West Midlands Multi-centre Research Ethics Committee and by the research and development department at each centre. The current version of the protocol can be found here: www.birmingham.ac.uk/secrab Oversight of the trial was provided by an independent Data Monitoring Committee.

### Patients

Patients with histological confirmed, invasive, early stage breast cancer with no evidence of metastatic disease were eligible for this study. Patients were required to have complete macroscopic excision of their tumour by mastectomy or breast conserving surgery. Additional eligibility criteria included; clear indication for adjuvant chemotherapy and radiotherapy, with adequate medical fitness including preserved cardiac, renal, hepatic and bone marrow function, and provision of written informed consent. Exclusion criteria included; prior chemotherapy, previous cancer, and pregnancy. No patients in the mastectomy group had immediate reconstruction however delayed reconstruction was allowed depending on patient and clinician choice.

### Randomisation and masking

Following informed consent eligible patients were randomised (1:1) to sequential treatment, chemotherapy followed by radiotherapy (control arm) or synchronous treatment, radiotherapy given concurrently or as a sandwich with chemotherapy (research arm). Randomisation was performed centrally by the Cancer Research UK Clinical Trials Unit (CRCTU) at the University of Birmingham using a computer generated random permuted block assignment. Stratification was performed according to centre, axillary surgery, chemotherapy regimen and inclusion of radiotherapy boost. Patients were also given the option of participating in quality of life, dose intensity and cosmesis sub-studies.

### Procedures

Patients were treated with adjuvant chemotherapy from protocol mandated regimens reflective of common practice at the time of recruitment. Permitted regimes were cyclophosphamide, methotrexate and 5-fluorouracil + folinic acid (CMF; intravenous or oral; six cycles), four cycles of anthracycline followed by four cycles of CMF, or Mitomycin-C, Mitoxantrone and Methotrexate (MMM). Approved chemotherapy schedules are listed in Table 1.

**Table 1:** Chemotherapy regimens and radiotherapy schedules.

Chemotherapy regimens	Dose (mg/m^2^)	Route	Frequency	Cycle duration (days)	Number of cycles
**CMF Regimens**					
*CMF “Classical”*					
Cyclophosphamide	100	Oral	D1−14	28	6
Methotrexate	40	IV	D1+8		
5-Fluorouracil	600	IV	D1+8		
+ Folinic acid: 15 mg oral 4 hourly × 6 doses 24 hours after methotrexate					

*CMF “Classical” IV*					
Cyclophosphamide	600	IV	D1+8	28	6
Methotrexate	40	IV	D1+8		
5-Fluorouracil	600	IV	D1+8		
+ Folinic acid: 15 mg oral 4 hourly × 6 doses 24 hours after methotrexate					

*CMF (6–8) IV 3-Weekly (Scottish Breast Group Schedule)*					
Cyclophosphamide	750	IV	D1	21	6–8
Methotrexate	50	IV	D1		
5-Fluorouracil	600	IV	D1		

**Anthracycline-containing Regimens**					
*3-Weekly Epirubicin/CMF (Scottish Breast Group Schedule)*					
Epirubicin	100	IV	D1	21	4
Followed CMF (6–8) IV 3-Weekly (Scottish Breast Group Schedule) for 4 cycles					

*Epirubicin + “Classical” CMF*					
Epirubicin, or Doxorubicin	100 75	IV IV	D1	21	4
Followed by CMF “Classical” oral or IV for 4 cycles					

*Bonadonna Regimen*					
Adriamycin	75	IV	D1	21	4
Followed IV 3-Weekly CMF for 4–8 cycles					

**Mitomycin-C, Mitoxantrone and Methotrexate**					
Mitoxantrone	8	IV	D1	21	6
Methotrexate	35	IV	D1		
Mitocycin-C	8	IV	D1		
+ Folinic acid: 15 mg oral 4 hourly × 6 doses 24 hours after methotrexate					

Radiotherapy Schedules	Dose (Gy)	Number of fractions	Duration (weeks)
	39	13	5
	40	15	3
	42.5 (chest wall only, no boost)	15	3
	45	20	4
	46	23	4 ½
	50	25	5

IV = intravenous; D = day

Radiotherapy practice was not standardised in the UK when this trial was running. However the Standardisation of Radiotherapy (START) trial was run in parallel to SECRAB [13]. Therefore, in those centres running both SECRAB and START Quality Assurance (QA) was applied as per START to the radiotherapy treatments during SECRAB. Those centres not recruiting to START had their own in-house QA protocol which was mandatory in the UK as part of the Quality of Assurance in Radiotherapy (QUART) guidance which came into place in 1993. Six widely used standard radiotherapy schedules were permitted ranging from 15 to 25 daily fractions (see table 1) with or without subsequent boost doses. Radiotherapy was allowed to the breast or chest wall, the axilla and supraclavicular fossa at the clinicians’ discretion. Both 2D, and 3D planning (once available), were permitted. Internal mammary node irradiation was not allowed. It was advised that patients with excessively large breast size should be excluded as they might be at greater risk of acute skin toxicity [14]. The recommended regime for synchronous therapy was 40 Gy in 15 fractions with optional boost, but other doses were allowed including 45 Gy in 20 fractions and 50 Gy in 25 fractions. When radiotherapy courses >3 weeks were given synchronously, radiotherapy was omitted on the day of chemotherapy administration. In anthracycline containing regimens, synchronous radiotherapy was given after the intravenous component of the first cycle of CMF, once anthracycline was completed. In CMF/MMM regimens the synchronous radiotherapy was delivered after the intravenous component of the second or third cycle of chemotherapy. For patients having a radiotherapy boost the subsequent CMF/MMM chemotherapy could be deferred by one week for patients treated in the synchronous arm. Hormone therapy was allowed at clinicians’ discretion. Patients were followed up annually for ten-years.

### Outcomes

The primary outcome measure defined in the protocol was local recurrence rates at five and ten years. Local recurrence was not defined in the protocol hence local recurrence (ipsilateral breast/chest wall only) defined as the time from randomisation to the earliest documentation of recurrence is reported as the primary outcome measure. In addition, loco-regional in-field recurrences (ipsilateral breast/chest wall, axilla and/or supraclavicular fossa) is also reported. The first site of recurrence was recorded for analysis but simultaneous or later loco-regional recurrences were noted. Secondary outcome measures in all patients were distant recurrence rates, survival (disease free survival and overall survival), along with acute toxicity causing significant treatment delay or dose reduction, and other late effects of treatment.

Radiotherapy-induced acute skin toxicity was graded according to the scoring system described previously by Fernando et al., as none, mild (mild/moderate erythema; no dry or moist desquamation), moderate (marked erythema, 5–10% dry of moist desquamation with complete healing within four weeks), or severe (dry or moist desquamation in >10% or skin reaction causing delay or incomplete healing or ulceration >4 weeks post-radiotherapy) [14]. This system shows simplified equivalence to the toxicity criteria of the Radiation Therapy Oncology Group (RTOG) in which moderate skin reaction would be completely healed within four weeks and severe skin toxicity would not be healed by four weeks [15]. At the time of trial initiation, lymphedema was not part of any late-effect scoring system, therefore we developed our own scoring system according to a protocol equivalent to the European Organisation for Research and Treatment of Cancer (EORTC) toxicity scale and National Cancer Institute (NCI) Common Toxicity Criteria (CTC) grading system with nil/mild being none or asymptomatic requiring no treatment, moderate being requiring treatment but reversible and severe being non-reversible and/or affecting limb function. This was analogous to grades 0/1, 2 and 3 later published by Burmeister et al. [16]. Telangiectasia was graded as minimal, moderate, or severe as previously described by Turesson and Thomas [17]. Other adverse reactions to radiotherapy were recorded as present or absent. All serious adverse events (SAEs) were recorded.

Outcome measures in the detailed sub-study included; reduction in dose intensity of chemotherapy, cosmetic result and quality of life. Changes in breast appearance (photographic) were scored by three observers blind to patient identity, treatment allocation, and year of follow-up, and a final agreed score reached by consensus as per the START trial [18]. Quality of life was measured using the EORTC QLQ-C30 (version 2) core questionnaire, the EORTC-QLQ BR23 (version 1) supplementary Breast Cancer module (BR23), and the Women's Health Questionnaire completed at baseline, on completion of chemo-radiotherapy, one, two, and five years following surgery.

### Statistical analysis

At study conception local recurrence rates were reported to be 5–10% [19]. Thus recruiting 2000 patients would detect a 4% difference with 80% power and 3% difference with 65% power at a 5% level of significance. To provide sufficient statistical power to perform the sub-group analyses, 1000 patients receiving each of the two principal chemotherapy regimens (CMF and anthracycline-CMF) were recruited into the study allowing detection of a 5% difference in local recurrence rates, with 80% power and a 5% level of significance. The chemotherapy regimen was chosen prior to randomisation and was also a stratification factor to ensure an equal distribution to each arm. Analyses were performed on the ‘intention to treat’ population which included all patients as randomised who provided consent. Differences in time to event outcomes between treatment arms were summarised using Kaplan-Meier plots and quantified as hazard ratios (HRs) with 95%-confidence intervals (CIs) along with P-values estimated using Cox proportional-hazards model with and without adjustments for stratification factors. The primary analysis (time to local recurrence) and time to loco-regional in-field recurrence are estimated using Cox proportional-hazards model and presented without adjustment for stratification factors. The sub-group analyses for anthracycline-CMF and CMF (local recurrence), distant recurrence, disease free survival and overall survival have all been estimated using Cox proportional-hazards model and presented with adjustment for stratification factors. Unplanned sub-group analyses were performed and Hazard Ratios (HRs) estimated via Cox proportional-hazards model with adjustment for stratification factors, and presented in a forest plot for patient and disease characteristics, and for treatment factors. Heterogeneity has been assessed using the I^2^ statistic. Chi-squared tests were used to compare the number of patients with moderate/severe toxicity versus none/mild across both treatment arms and patients who have and have not experiences a specific toxicity. In addition, a planned comparison of toxicity between the treatment arms was adjusted for radiotherapy schedule (three weeks versus > 3 weeks).

Analyses were performed using Stata 15.1.

### Role of the funding source

Funding for SECRAB was provided by Cancer Research UK. A small educational grant was provided by Pharmacia for collection of photographs. The funders of the study had no role in study design, data collection, data analysis, data interpretation, or writing of the report. The corresponding author had full access to all the data in the study. The corresponding author had final responsibility for the decision to submit for publication.

## Results

**Fig. 1:**
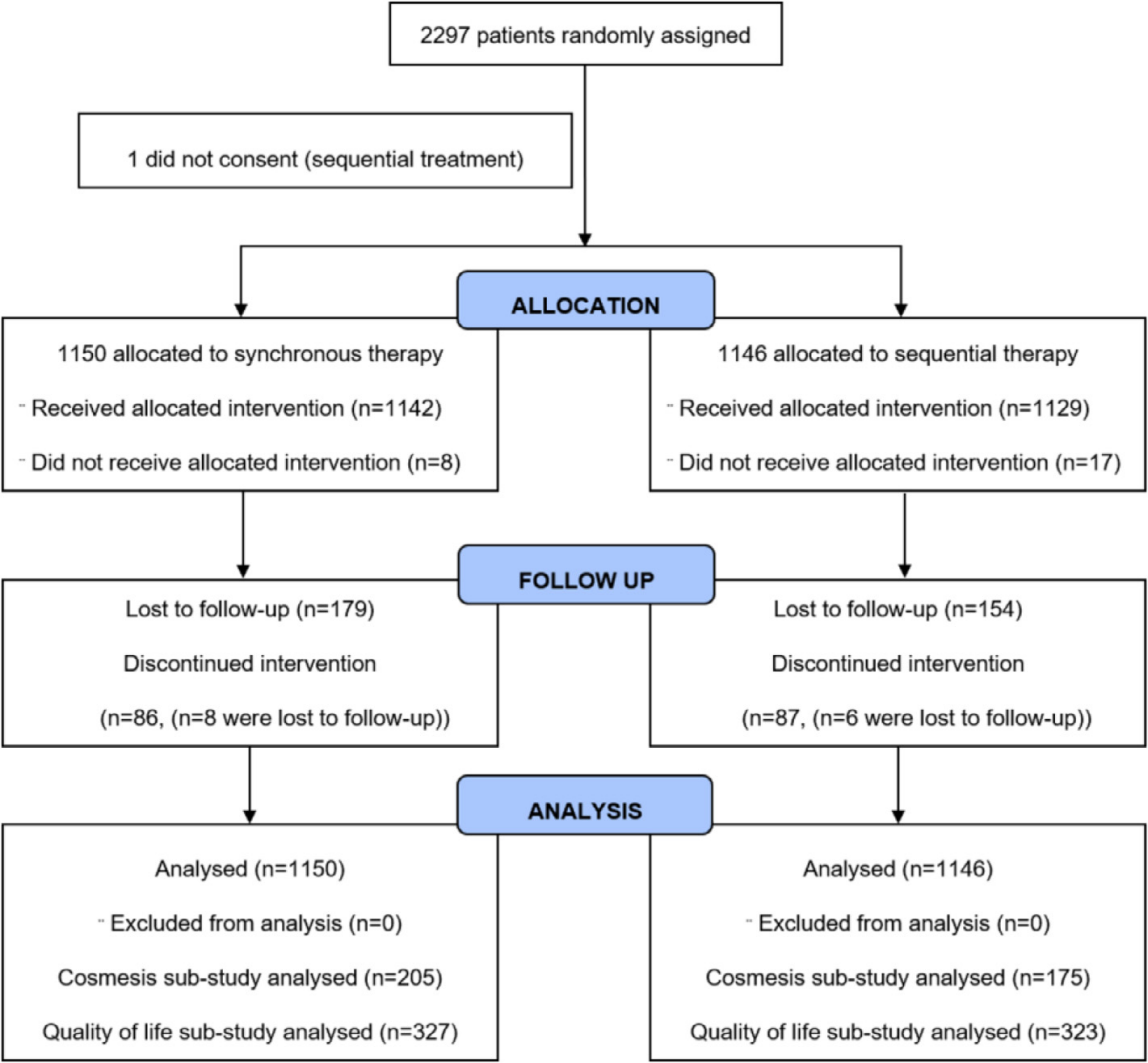
SECRAB trial profile. The following patients did not receive allocated intervention: synchronous therapy – CT not given *n* = 2, RT not given *n* = 6; sequential therapy – CT not given *n* = 0, RT not given *n* = 17. Discontinued intervention refers to participants who discontinued the trial treatment (synchronous therapy – withdrawn from CT *n* = 79, withdrawn from RT *n* = 7; sequential therapy – withdrawn from CT *n* = 82, withdrawn from RT *n* = 5) or were lost to follow-up while on treatment (synchronous therapy – *n* = 8; sequential therapy – *n* = 6).

Between 02-July-1998 and 25-March-2004, 2297 patients were recruited into the trial, 1150 were assigned to the synchronous arm and 1146 to the sequential arm (Fig. 1). One patient was subsequently found to have not provided full informed consent and was excluded. Baseline characteristics were evenly balanced between the two arms (Table 2). The majority of patients were node positive (1422/2296, 61.9%) with 538 (23.4%) of 2296 patients having more than four involved axillary lymph nodes and 1312 (57.1%) patients having grade 3 disease.

**Table 2:** Patient characteristics.

	Synchronous	Sequential	Total
	N = 1150	N = 1146	N = 2296
**Patient baseline characteristics, n (%)**			
*Age (years)*			
Median	52	51	51
Interquartile range	45–58	45–58	45 – 58
Range	24–77	24–79	24 – 79
<50	481 (41.8)	493 (43.0)	974 (42.4)
≥50	669 (58.2)	653 (57.0)	1322 (57.6)

*Type of Surgery*			
Mastectomy	514 (44.7)	497 (43.4)	1011 (44.0)
Wide Local Excision	625 (54.3)	643 (56.1)	1268 (55.2)
Other	11 (1.0)	6 (0.5)	17 (0.8)

*Vascular/Lymphatic Invasion*			
Not seen	624 (54.2)	621 (54.2)	1245 (54.2)
Present	523 (45.5)	521 (45.5)	1044 (45.5)
Unknown	3 (0.3)	4 (0.3)	7 (0.3)

*Number of Nodes*			
Negative	428 (37.2)	444 (38.7)	872 (38.0)
1–3 positive	444 (38.6)	440 (38.4)	884 (38.5)
4 + positive	277 (24.1)	261 (22.8)	538 (23.4)
Missing	1 (0.1)	1 (0.1)	2 (0.1)

*Tumour Grade*			
Grade 1 – Well differentiated	76 (6.6)	68 (5.9)	144 (6.3)
Grade 2 – Moderately differentiated	415 (36.1)	411 (35.9)	826 (36.0)
Grade 3 – Poorly differentiated	654 (56.9)	658 (57.4)	1312 (57.1)
Unknown	5 (0.4)	9 (0.8)	14 (0.6)

*Tumour Size (mm)*			
N	1136	1137	2273
Median	22	22	22
Interquartile range	16–30	16–30	16–30
Range	2–100	2–210	2–210

*ER Status*			
Negative	401 (34.9)	387 (33.8)	788 (34.3)
Positive	703 (61.1)	724 (63.2)	1427 (62.2)
Unknown	46 (4.0)	35 (3.0)	81 (3.5)

*PgR Status*			
Negative	250 (21.7)	255 (22.5)	505 (22.0)
Positive	265 (23.1)	258 (22.7)	523 (22.8)
Unknown	635 (55.2)	623 (54.8)	1268 (55.2)

*HER2 Status*			
Negative	104 (9.0)	122 (10.7)	226 (9.9)
Positive	39 (3.4)	44 (3.8)	83 (3.6)
Unknown	1007 (87.6)	980 (85.5)	1987 (86.5)

*Present Menopausal Status*			
Pre	400 (34.8)	408 (35.6)	808 (35.2)
Peri	91 (7.9)	95 (8.3)	186 (8.1)
Post	524 (45.6)	515 (44.9)	1039 (45.2)
Unknown	135 (11.7)	128 (11.2)	263 (11.5)

**Endocrine Therapy**			
*Ovarian ablation*			
No	1077 (93.6)	1063 (92.8)	2140 (93.2)
Yes	64 (5.6)	75 (6.5)	139 (6.1)
Unknown	9 (0.8)	8 (0.7)	17 (0.7)

*Tamoxifen*			
No	363 (31.6)	353 (30.8)	716 (31.2)
Yes, with CT	319 (27.7)	295 (25.7)	614 (26.7)
Yes, after CT	454 (39.5)	490 (42.8)	944 (41.1)
Unknown	14 (1.2)	8 (0.7)	22 (1.0)

*Other Hormone Manipulation*			
No	1116 (97.0)	1121 (97.8)	2237 (97.4)
Yes	24 (2.1)	15 (1.3)	39 (1.7)
Unknown	10 (0.9)	10 (0.9)	20 (0.9)

A larger proportion of patients received CMF (1244/2296, 54.2%) chemotherapy than anthracycline containing treatment (1041/2296, 45.3%). Of the patients receiving anthracycline containing regimens, 1011 received epirubicin-CMF, with 30 patients receiving either a Bonadonna regimen of Adriamycin-CMF or a variant. Nine (0.4%) patients received MMM. Two patients (0.1%) did not receive chemotherapy. A total of 2258 (98.3%) of 2296 patients received radiotherapy. The majority of patients received 40 Gy in 15 fractions over three weeks (1392/2296, 60.6%) or 42.5Gy in 15 fractions over three weeks with no boost (343/2296, 14.9%), with the remainder receiving schedules >3 weeks (523/2296, 22.8%). The treatment delivered was balanced between the arms, including patients receiving radiotherapy boost (Table 3).

**Table 3:** Treatments delivered.

	Synchronous	Sequential	Total
	N = 1150	N = 1146	N = 2296
**Treatment delivered**			
*Chemotherapy*+			
CMF	617 (53.7)	627 (54.7)	1244 (54.2)
Anthracycline-CMF	525 (45.7)	516 (45.0)	1041 (45.3)
Other	6 (0.5)	3 (0.3)	9 (0.4)
Not given	2 (0.2)	0 (0.0)	2 (0.1)

*Radiotherapy*+			
39 Gy in 13 fractions over 5 weeks	10 (0.9)	8 (0.7)	18 (0.8)
40 Gy in 15 fractions over 3 weeks	706 (61.4)	686 (59.9)	1392 (60.6)
42.5Gy in 15 fractions, 3 weeks, no boost	174 (15.1)	169 (14.7)	343 (14.9)
45 Gy in 20 fractions over 4 weeks	73 (6.3)	68 (5.9)	141 (6.1)
46 Gy in 23 fractions over 4 ½ weeks	69 (6.0)	76 (6.6)	145 (6.3)
50 Gy in 25 fractions over 5 weeks	108 (9.4)	111 (9.7)	219(9.5)
Radiotherapy not given	6 (0.5)	23 (2.0)	29 (1.3%)
Missing	4 (0.3)	5 (0.4)	9 (0.4)

*Boost Given*+			
No	803 (69.8)	762 (66.5)	1565 (68.2)
Yes	340 (29.6)	360 (31.4)	700 (30.5)
Radiotherapy not given	6 (0.5)	23 (2.0)	29 (1.3%)
Missing	1 (0.1)	1 (0.1)	2 (0.1)

*Time from Surgery to Radiotherapy, weeks (interquartile range)*			
CMF	12 (10–15)	30 (27–33)	21.5 (12–30)
Anthracycline + CMF	19 (18–21)	34 (31–36)	26 (19–34)
MMM	11.5 (10–12)	27 (25–38)	12 (11–25)

+ This table shows the treatment actually received instead of treatment intent.

**Fig. 2:**
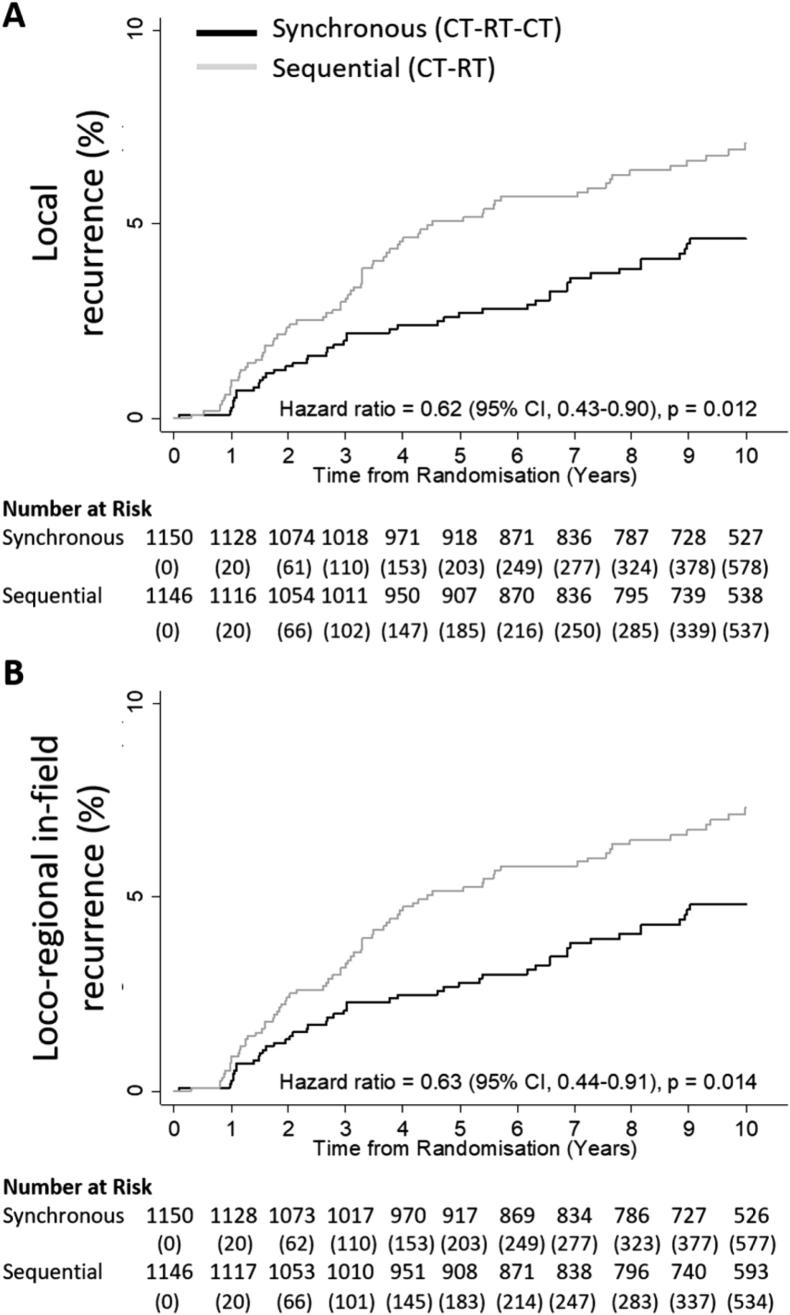
Kaplan–Meier 10-year analyses of primary outcome measures. Panel A shows the primary outcome of local tumour recurrence rates, defined as the time from randomisation to the earliest documentation of local recurrence. Panel B shows the primary outcome of loco-regional in-field recurrence rates, defined as the time from randomisation to the earliest documentation of loco-regional in-field recurrence. Patients were censored at date of death or date last seen. The Kaplan-Meier function (1-survival time) has been plotted for the above panels. The unadjusted hazard ratios, 95% confidence intervals and p-values were derived from Cox regression models. CI = Confidence Interval.

With a median follow-up of 10.2 years (IQR: 9.7, 10.5), local relapses were observed in 45 (3.9%) of the 1150 patients randomised to synchronous therapy and 72 (6.3%) of 1146 patients assigned to sequential therapy (Table 4). The five-year local recurrence rate was 2.7% (95% CI: 1.9–3.9) in synchronous arm and 5.1% (95% CI: 3.9–6.6) in the sequential arm, similar results were seen for the loco-regional in-field recurrence rates (Table 4). Ten-year local recurrence rates were 4.6% and 7.1% in the synchronous and sequential arms respectively, with a significant benefit of synchronous treatment, with a HR of 0.62 (95% CI: 0.43–0.90; *p* = 0.012) (Fig. 2A), and also loco-regional in-field recurrences, with a HR of 0.63 (95% CI: 0.44–0.91; *p* = 0.014) (Fig. 2B). These results are consistent with the adjusted results. Table 4 also summarises the distribution of cases depending on whether they first recurred locally, regionally or both together. Six case (two in the synchronous arm and four in the sequential) occurred in both the breast/chest wall and regional lymph nodes at the same time.

**Table 4:** Local recurrence rates based on first site of occurrence.

	Synchronous	Sequential	
	N = 1150	N = 1146	
*Local Recurrence Rates, % (CI)*			p-value
Local recurrence			0.012
5-year	2.7 (1.9-3.9)	5.1 (3.9–6.6)	
10-year	4.6 (3.5-6.2)	7.1 (5.7–8.9)	
Loco-regional in-field recurrence			0.014
5-year	2.8 (2.0-4.0)	5.2 (4.0–6.7)	
10-year	4.8 (3.6-6.4)	7.3 (5.8–9.1)	

*Local and Regional In-field Recurrences, n*			Total
Local recurrence alone	42 (3.7)	64 (5.6)	106 (4.6)
Local recurrence with a regional in-field recurrence	2 (0.2)	4 (0.3)	6 (0.3)
Regional in-field recurrence without a local recurrence	3 (0.3)	6 (0.5)	9 (0.4)
Loco-regional in-field recurrences	47 (4.1)	74 (6.5)	121 (5.3)

P values were calculated by the Cox proportional-hazards model.

**Fig. 3:**
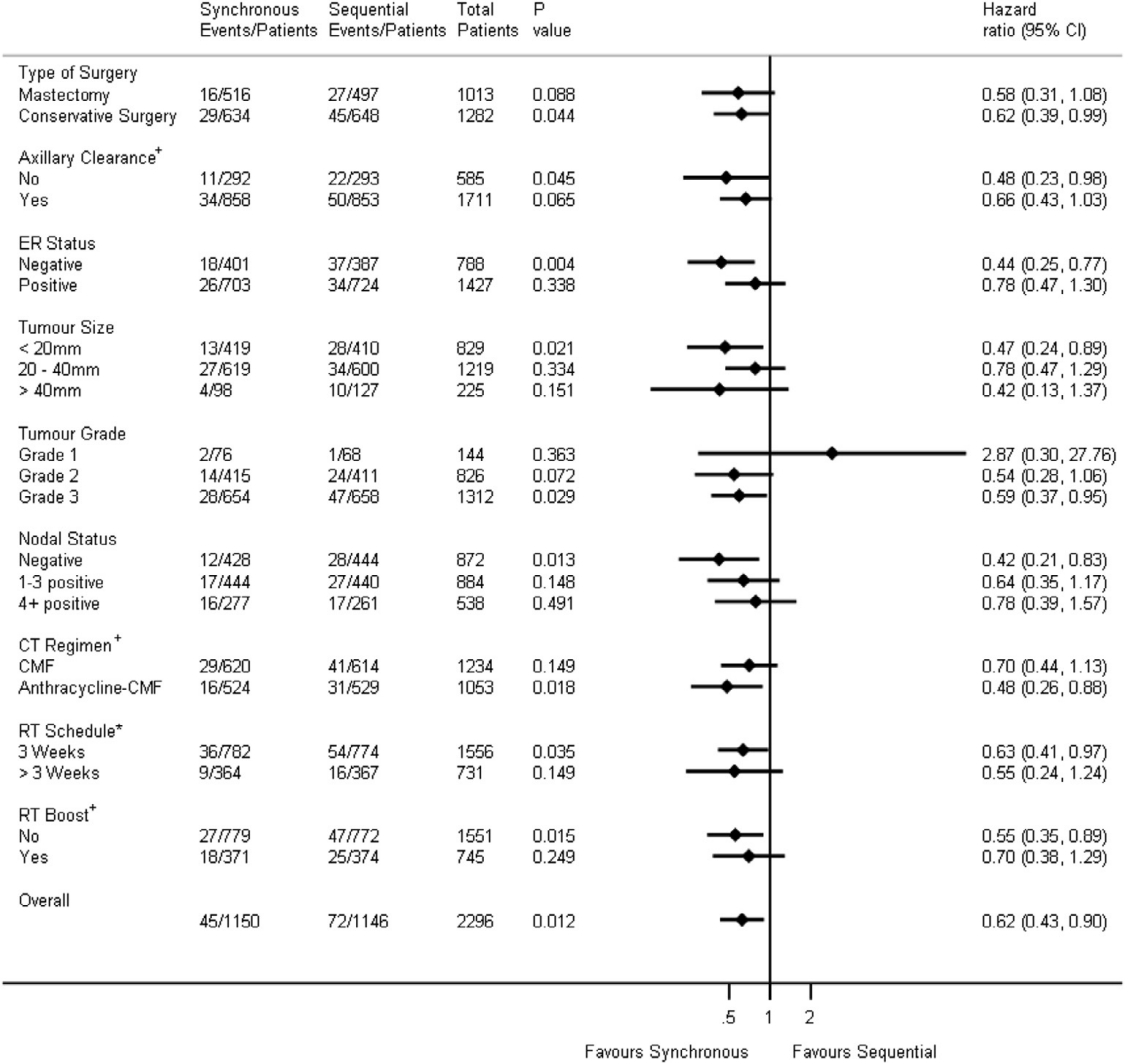
Forest plot of local recurrence according to sub-groups. The solid vertical line represents the null hazard ratio value of 1. Horizontal lines represent confidence intervals with hazard ratios indicated by diamonds. Conservative surgery includes wide local excision and quadrantectomy. RT schedules are based on treatment intent at randomisation. Chemotherapy regimen is based on treatment intent at randomisation and it is presented this way as it is powered for in the study. ^+^ shows stratification variables. Chemotherapy regimen, radiotherapy schedule and radiotherapy boost were based on intent to treat not actual treatment given. * 3 weeks includes the following radiotherapy schedules: 40 Gy in 15 fractions delivered over 3 weeks and 42.5 Gy in 15 fractions delivered over 3 weeks; >3 weeks includes the following radiotherapy schedules: 39 Gy in 13 fractions delivered over 5 weeks, 46 Gy in 23 fractions delivered over 4 ½ weeks, 45 Gy in 20 fractions delivered over 4 weeks and 50 Gy in 25 fractions delivered over 5 weeks. CI = Confidence Interval.

A forest plot of local recurrence according to sub-groups is presented in Fig. 3. Although no evidence of heterogeneity was observed, the results indicate a trend towards benefit of synchronous chemo-radiotherapy throughout, other than for grade 1 tumours (which included only three events).

In those patients treated with an anthracycline-CMF regimen, the ten-year local relapse rates for synchronous and sequential patients were 3.5% versus 6.7% respectively, with a HR 0.48 (95% CI: 0.26–0.88; *p* = 0.018). While for CMF, the ten-year local relapse rates of 5.6% versus 7.5% for synchronous and sequential patients respectively were observed, which was not significant (HR 0.70 (95% CI: 0.44–1.13; *p* = 0.149)).

**Fig. 4:**
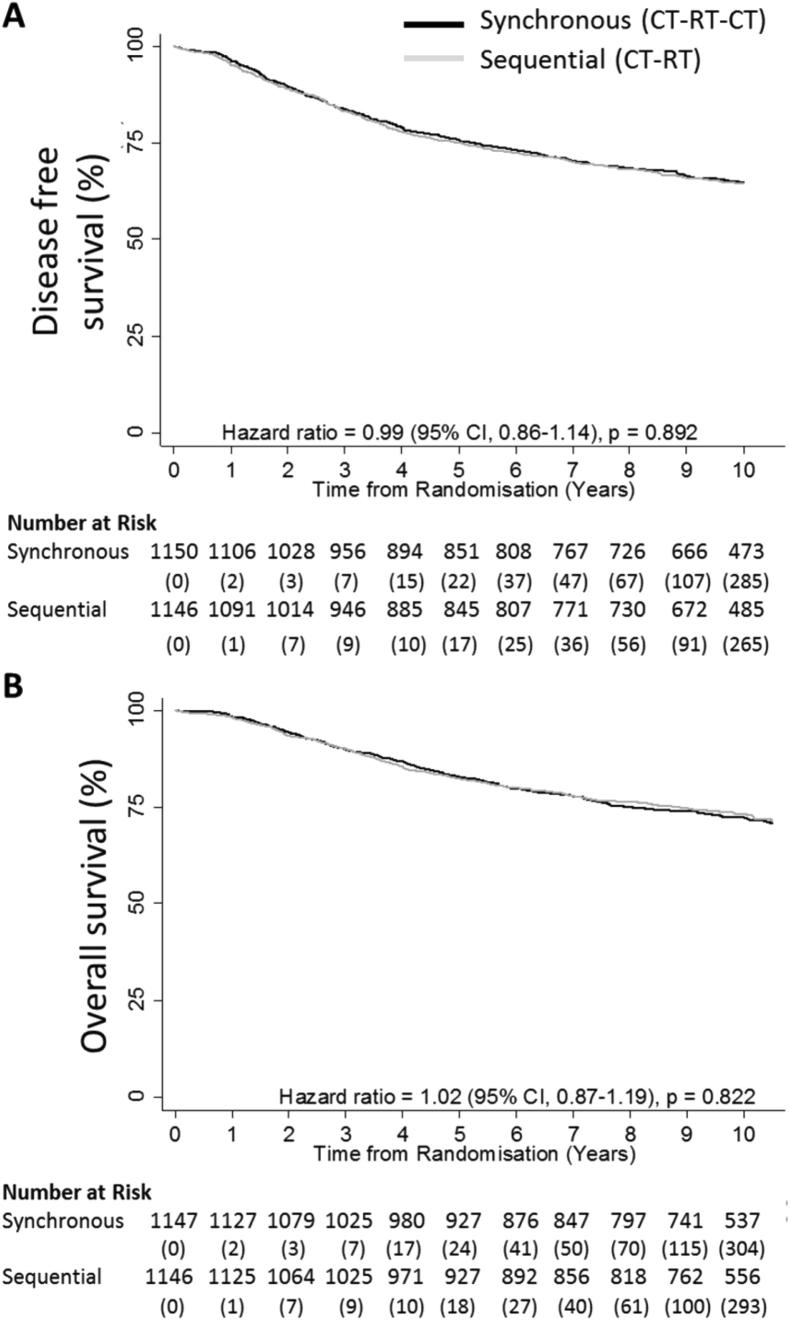
Kaplan–Meier ten-year analyses of secondary outcome measures. Panel A shows disease free survival defined as the time from randomisation to the earliest documentation of any recurrence or death. Panel B shows overall survival defined as the time from randomisation to death from any cause. Patients were censored at date last seen. The adjusted hazard ratios, 95% confidence intervals and p-values were derived from Cox regression models. CI = Confidence Interval.

The ten-year distant recurrence rate of 25.5% in the synchronous arm versus 24.6% in the sequential arm with an HR of 1.04 (95% CI: 0.88–1.22; *p* = 0.68). Disease free survival and overall survival were not statistically different between the two arms. The ten-year disease free survival rate was 64.9% in the synchronous arm (404 recurrences) and 64.6% in the sequential arm (406 recurrences) with HR 1.00 (95% CI: 0.87–1.14; *p* = 0.951) (Fig. 4A). The ten-year overall survival rate was 72.4% in the synchronous arm with a total of 316 deaths from any cause and 73.4% in the sequential arm with 312 deaths; HR 1.02 (95% CI: 0.87–1.19; *p* = 0.824) (Fig. 4B). In an unplanned exploratory sub-group analysis for patients receiving anthracycline-CMF the ten-year disease free survival rate was 67.7% in the synchronous arm compared to 63.5% in the sequential arm (HR = 0.86 (95% CI: 0.70–1.06; *p* = 0.170)). Furthermore, the overall survival rate for this unplanned exploratory sub-group analysis was 75.8% in the synchronous arm compared to 72.3% in the sequential arm (HR = 0.85 (95% CI: 0.66–1.08; *p* = 0.171)).

Of those patients who received any modification to their treatment plan, 626 were due to chemotherapy-related toxicities. The most common reasons were myelosuppression (140 (12.2%) in the synchronous arm, 98 (8.6%) in the sequential (*p* = 0.004)), oral gastrointestinal toxicities (66 (5.7%) in the synchronous arm, 78 (6.8%) in the sequential (*p* = 0.297)) and infection (61 (5.3%) in the synchronous arm, 57 (5.0%) in the sequential (*p* = 0.713)). Of those patient whose radiotherapy delays were >7 days, more were observed in the synchronous arm compared to the sequential arm (1.0% (*n* = 12) versus 0.3% (*n* = 3); *p* = 0.02). Only two of which were attributed to neutropenia, both occurring in the synchronous arm. However these numbers were probably too small to be of clinical relevance.

Although differences in the synchronous arm were observed, importantly, there was no significant difference in dose reduction of >20% between the synchronous and sequential arms. This was confirmed in the detailed dose intensity sub-study analysis; 372 (88.4%) of 421 patients in the synchronous arm received >85% dose intensity of chemotherapy, compared to 369 (90.0%) of 410 of patients in the sequential arm [20].

Acute skin toxicity was more common with synchronous radiotherapy; 275 (24.0%) patients developed either a moderate or severe acute skin reaction with synchronous treatment compared to 166 (14.8%) for those treated with sequential therapy (*p* < 0.0001). Of these, 44 (3.8%) patients had a severe reaction in the synchronous arm compared to 12 (1.1%) treated sequentially. However an unplanned exploratory analysis by radiotherapy schedule showed that patients receiving >3 weeks of radiotherapy had a significantly worse acute skin reaction than those receiving three weeks (24.5% versus 16.7%; *p* < 0.0001). There was an increase in moderate/severe telangiectasia of the treated area in the synchronous arm compared to the sequential arm (35/1150 (3.0%) versus 19/1146 (1.7%), respectively; *p* = 0.03). No statistical difference in telangiectasia was seen in those patients treated using a three-weekly schedule comparing synchronous to sequential (19/782 (2.4%) versus 9/774 (1.2%), respectively; *p* = 0.06).

No evidence of a difference was observed in any other effects including lymphedema, subcutaneous fibrosis, rib fracture, symptomatic acute and late pneumonitis, ischaemic heart disease, breast shrinkage, or brachial plexopathy (table 5). Detailed patient reported outcomes including cosmesis and global quality of life showed no difference over a five-year period [21], [22].

**Table 5:** Acute and late radiotherapy toxicities.

	Synchronous	Sequential	
	N = 1150	N = 1146	
*Acute Toxicity, n (%)*			P-value
In-field skin toxicity			<0.001*
None	262 (22.9)	410 (36.5)	
Mild	599 (52.4)	544 (48.4)	
Moderate	231 (20.2)	154 (13.7)	
Severe	44 (3.8)	12 (1.1)	
Unknown	8 (0.7)	3 (0.3)	
Pneumonitis (Acute)	5 (0.4)	1 (0.1)	0.11

*Late Toxicity, n (%)*			
Moderate/severe lymphedema	78 (6.8)	65 (5.7)	0.27
Severe subcutaneous fibrosis	17 (1.5)	11 (1.0)	0.26
Moderate/severe telangiectasia	35 (3.0)	19 (1.7)	0.03
Ischaemic heart disease	7 (0.6)	3 (0.3)	0.28
Rib fracture	7 (0.6)	5 (0.4)	0.50
Symptomatic lung fibrosis	5 (0.4)	3 (0.3)	0.69
Pneumonitis	1 (0.1)	1 (0.1)	1
Brachial plexopathy	2 (0.2)	2 (0.2)	1

P values were calculated by the Chi-squared test. *P-value for in-field skin toxicity compares patients with moderate/severe toxicity versus none/mild across both treatments. P-value for late toxicities compares patients with moderate/severe versus none/mild and also those patients who have or have not experienced a specific toxicity across both treatment arms.

There were 255 Serious Adverse Events (SAEs) reported in the trial, 123 on the synchronous and 132 on the sequential arm. Myelosuppression and subsequent neutropenic sepsis were the most common SAEs related to chemotherapy, noted in 117/2296 (5.1%) patients. Of these, 62 patients received synchronous treatment and 55 patients received sequential treatment. An SAE was reported for one patient, on the sequential arm who received CMF, who developed myelodysplasia approximately two years after the completion of radiotherapy. This progressed to Acute Myeloid Leukaemia from which the patient subsequently died.

Thirteen SAEs were reported as related to radiotherapy, of which seven events (six patients) were regarded, on review by the Chief Investigator, as being related to the radiotherapy reaction. Five patients in the synchronous arm had severe acute skin desquamation which required hospitalisation. Four out of the five made a complete recovery with full healing. Three of these patients had been treated with a >3 weeks radiotherapy schedule. One patient (two SAEs) developed a chronic ulcer in the inframammary fold which required surgical intervention. This patient had a significant breast overhang.

## Discussion

This is the largest trial to date reporting the role of adjuvant synchronous chemo-radiotherapy in the management of breast cancer. Ten-year follow up concludes that synchronous chemo-radiotherapy significantly improves local recurrence rates with a 38% reduction in risk of local recurrence (HR 0.62; 95% CI: 0.43–0.90; *p* = 0.012). This was delivered with an acceptable modest increase in acute toxicity. A trend towards benefit of synchronous chemo-radiotherapy can be seen throughout all subgroups (Fig. 3), with some groups appearing to receive a greater treatment benefit. We note that a lack of statistical power is inherent in such analyses. Furthermore we acknowledge that no formal measure has been taken to address the issue of multiple testing. We also reiterate that the analysis of the anthracycline-CMF chemotherapy question was planned and appropriately powered.

Avoiding local recurrence has enormous psychological and physical benefits for the patient including avoiding the need to undergo further surgery. The observed reduction in risk of local and loco-regional recurrence is particularly pertinent as the results of the 2011 Early Breast Cancer Trialists' Collaborative Group (EBCTCG) overview showed that one life could be saved for every four recurrences prevented for patients treated by conservative surgery and radiotherapy [19]. Furthermore, during the 2014 EBCTCG overview, it was also demonstrated that for patients treated by mastectomy the benefit might be even larger for those with one-to-three involved lymph nodes where they suggested one breast cancer death was avoided for every 1.5 recurrences of any type [23]. We do however note this cannot be generalised to all node-positive patients especially in those patients with more than four positive lymph nodes [24] supporting the data presented in Fig. 3.

The SECRAB trial was not powered for a survival outcome. However we note a non-significant 4.2% advantage in disease free survival and a 3.5% advantage in overall survival were seen in the synchronous arm for patients treated with anthracycline-CMF. These improvements are consistent with the results of the EBCTCG overviews [19], [23].

The greatest benefit of synchronous chemo-radiation was in patients treated with anthracycline-CMF even though these patients would have had a greater delay in starting radiotherapy compared to those patients treated with CMF. This was a surprising result as radiotherapy was administered earlier in the synchronous arm with CMF compared to anthracycline-CMF. It is the delivery of concurrent treatment which is of paramount importance. In fact anthracycline-based chemotherapy would now be considered standard treatment for patients with early breast cancer. The local recurrence rates, in keeping with many recently reported trials [25], were lower than originally expected, but for high risk patients with nodal involvement or ER negative tumours there would be a substantial benefit [26].

There was an increase in acute skin toxicity, telangiectasia and myelosuppression in patients treated with synchronous treatment, but other late effects including pneumonitis, fibrosis, rib fracture, lymphedema, brachial plexopathy, and cardiac events were similar in both trial arms. We note that a large cohort such as ours can detect very small differences. A greater proportion of patients with moderate or severe acute skin reactions had been treated with radiotherapy schedules >3 weeks and no significant difference in telangiectasia was seen in those patients treated using a three-weekly schedule. These data are consistent with the results of the START trial [27]. It is therefore recommended that synchronous chemo-radiotherapy should not be used in patients with large breast size where concerns may be present with regard to acute skin toxicity [14]. Although we note, with modern forward planned intensity modulated radiotherapy (IMRT), patients with larger breast size would have more conformal treatment and less acute skin toxicity. We would also advise that patients being treated with synchronous chemo-radiotherapy should be treated with a three-weekly radiotherapy regimen to avoid additional acute skin toxicity. Apart from the significant improvement in local/loco-regional control rates, a further advantage of synchronous treatment is that it shortens the overall treatment time for the patient as they would complete adjuvant treatment at the end of their last chemotherapy cycle as opposed to waiting to start radiation following chemotherapy as is standard practice.

The SECRAB trial results are primarily applicable to CMF and anthracycline–CMF containing regimes which were the standard UK chemotherapy regimens between 1998 and 2004. However, the use of taxane (T) chemotherapy schedules are now common place in early breast cancer. These schedules are problematic in terms of synchronous radiotherapy administration due to the risk of acute pulmonary toxicity [28], although it should be noted that the TACT trial showed no significance difference in disease free survival between epirubicin-CMF and FEC-T arms [25]. It will therefore be pertinent to evaluate alternative regimens such as, E-T-CMF which has been shown to have an improved disease free survival compared to anthracycline-CMF [29]. They would still have the advantage that synchronous chemo-radiotherapy could be used as part of the treatment protocol as it can be given during the CMF part of the regimen. In addition, these findings may be translatable to other hypofractionated schedules [30]. For example, SECRAB may be compatible with dose dense chemotherapy which has been shown to produce a survival advantage over conventional chemotherapy particularly in the ER negative patients [31]. Some of these regimens include CMF such as used by Baldini et al., and Kummel et al. [32], [33]. Finally, it is worth noting that the radiotherapy techniques used in this trial predate computed tomography planning, image modulated radiotherapy and other techniques such as breath-hold which can reduce skin, lung and cardiac toxicity, respectively. It may be pertinent to re-evaluate the role of synchronous chemo-radiotherapy using these advanced technologies with more standard chemotherapy schedules in further randomised studies.

In summary, the results of this study suggest that for any patient having anthracycline-CMF, radiotherapy should be given synchronously between the first and second cycle of CMF using a three week fractionation unless the patient has an excessively large breast size.

## Contributors

INF (Chief Investigator and first author): conception and design of the study, interpretation of data, drafting and review of paper. SJB: study design, acquisition of data, interpretation of data, drafting and review of paper. KH: interpretation of data, drafting and review of paper. CLB: senior trial statistician, analysis of data, interpretation of data, and review of paper. IA: trial statistician, analysis of data, interpretation of data, drafting and review of paper. AM: independent senior statistician, interpretation of data, and review of paper. JD: professor of statistics responsible for the original design of the trial, member of the Trial Steering Committee, and review of paper. RG, MC, DS, RKA, AMB, AS, AG, PC, CJP and DWR: member of Trial Steering Committee, design of the study, acquisition of data, interpretation of data, and review of paper. JB, DR, TNL: acquisition of data, interpretation of data, and review of paper.

## Declaration of interests

INF and SJB received grants from Cancer Research UK and Pharmacia pertaining to this research but have no other competing interests. All other authors declare no competing interests.
