# Supplementary material for: Synchronous versus sequential chemo-radiotherapy in patients with early stage breast cancer (SECRAB): A randomised, phase III, trial
Source: Radiother Oncol. 2020 Jan;142:52–61. doi: 10.1016/j.radonc.2019.10.014 (PMC7005671; doi:10.1016/j.radonc.2019.10.014)
Supplement: Supplementary data 1 [file mmc1.docx]

**Supplementary** **Appendix**

The SECRAB trial investigators include the following:

Steering Committee - Rajiv Agrawal (Shrewsbury and Telford Hospital NHS Trust, Shrewsbury, UK), Sarah Bowden (University of Birmingham, Birmingham, UK [UoB]), Murray Brunt (University Hospitals of North Midlands and Keele University, Staffordshire, UK), Cassandra Brookes (Leicester University, UK), Peter Canney (Beatson West of Scotland Cancer Centre, Glasgow, UK), Mark Churn (Worcestershire Acute Hospitals NHS Trust, Worcestershire, UK), Indrajit Fernando (University Hospitals Birmingham NHS Foundation Trust Birmingham, UK [UHB],), Andrew Goodman (Torbay and South Devon NHS Foundation Trust, UK), Robert Grieve (University Hospital of Coventry and Warwickshire NHS Trust, Coventry, UK), Mike Hallissey (UHB), Andreas Makris (The Hillingdon Hospitals NHS Foundation Trust, Middlesex, UK), Ujjal Mallick (The Newcastle upon Tyne Hospitals NHS Foundation Trust, Newcastle Upon Tyne, UK), Andrea Marshall (University of Warwick, Warwick, UK), Susan O'Reilly (The Clatterbridge Cancer Centre NHS Foundation Trust, Merseyside, UK) Christopher Poole (University Hospital of Coventry and Warwickshire NHS Trust, Coventry, UK), Daniel Rea (UoB), Ann Robinson (Southend University Hospital NHS Foundation Trust, Essex, UK), Peter Simmonds (University Southampton Hospital NHS Foundation Trust, Southampton, UK), David Spooner (UHB), Jane Steven (UHB), Andrea Stevens (UHB).

Centre and Investigators (accrual): Alexandra Hospital: Dr C Irwin (5); Ayr Hospital: Dr D Ritchie (8); Beatson West of Scotland Cancer Centre: Prof C Twelves, Dr J Wallace, Dr H Yosef, and Dr F Yuille (123); Birmingham Heartlands Hospital: Dr I N Fernando (57); Bristol Haematology Oncology Centre: Dr J M Tomlinson and Dr E C Whipp (3); City Hospital: Dr D W Rea, and Dr D Spooner (64); Clatterbridge Cancer Centre: Dr P Clark, Dr E Errington, Dr A Flavin, Dr S O’Reilly, Dr A Sun-Myint, Dr I Syndikus, and Dr N Thorp (94); County Hospital: Dr A M Brunt and Dr D Fairlamb (3); Diana Princess of Wales Hospital: Dr P Mack (39); Dumfries & Galloway Royal Infirmary: Dr T Evans and Prof I Kunkler (23); For the Valley Royal Hospital: Dr G Fraser and Dr P Canney (13); Glan Clwyd Hospital: Dr A Champion and Dr J Bishop (41); Good Hope Hospital: Dr A Stevens and Dr T Latief (74); Hairmyres Hospital: Dr P Canney, Dr D Ritchie, and Dr H Yosef (9); Kidderminster General Hospital: Dr M Churn and Dr I Irwin (41); Luton and Dunstable Hospital: Dr A Makris, and Dr Ah-See (7); Manor Hospital: Dr I N Fernando (229); Mount Vernon Hospital: Dr A Makris and Dr P Ostler (14); New Cross Hospital: Dr R Allerton, Dr C Brammer, Dr M Churn, Dr D Fairlamb, Dr R Mehra, and Dr B Smith (94); Freeman Hospital: Dr P Dawes, Dr U K Mallick, and Dr W Taylor (9); Princess Royal Hospital: Dr R K Agrawal (5); Priory Hospital: Dr A Goodman, Dr D W Rea, and Dr A Stevens (8); Queen Elizabeth Hospital, Birmingham: Mr D England, Dr I N Fernando, Dr A Goodman, Dr T N Latief, Dr C J Poole, Dr D W Rea, Dr D Spooner, and Dr A Stevens (128); Queen Elizabeth Hospital, Gateshead: Dr H Lucraft (1); Queen’s Hospital: Dr A D Chetiyawardana and Dr P Chakraborti (44); Royal Devon & Exeter Hospital: Dr A Goodman and Dr A Hong (34); Royal Preston Hospital: Dr S Kumar (1); Royal Shrewsbury Hospital: Dr R K Agrawal (117); Royal South Hants Hospital: Dr A Last, Dr M Murray, and Dr P Simmonds (44); Royal Stoke University Hospital: Dr A Ali-Niaimi, Dr A M Brunt, Dr A M Cook, and Dr J Scoble (135); Russells Hall Hospital: Dr R Allerton (49); Sandwell General Hospital: Dr D Spooner (62); Selly Oak Hospital: Dr A Goodman, Dr TN Latief, Dr C J Poole, and Dr D W Rea (22); Singleton Hospital: Dr T Joannides, Prof R C F Leonard, and Dr M Rolles (70); Solihull Hospital: Dr A Stockdale and Dr M Tsalic (36); Southampton General Hospital: Dr A Robinson and Dr C Trask (3); Southend University Hospital: Dr W A Ella, Dr A Robinson, and Dr C Trask (71); Southport and Formby District General Hospital: Dr K Hayat and Dr A Sun-Myint (2); St. Bartholomew’s Hospital: Dr P Wells(5); Sunderland General Hospital: Dr U K Mallick (8); Torbay Hospital: Dr A Goodman and Dr A Hong (15); University Hospital, Coventry: Prof R Grieve, Dr C Irwin, Dr A Stockdale; and Dr J Worlding (328); University Hospital Crosshouse: Dr D Ritchie (40); Warrington Hospital: Dr I Syndikus (6); Western General Hospital: Prof I Kunkler (24); Weston General Hospital: Dr M J Tomlinson (1); Worcester Royal Infirmary: Dr A Goodman, Dr C Irwin and Dr A Stevens (25); Wrexham Maelor Hospital: Dr A Champion and Dr W M Soe (37); Ysbyty Gwynedd: Dr J Bishop (26)
